# Chiropractic and children: Is more research enough?

**DOI:** 10.1186/1746-1340-18-11

**Published:** 2010-06-02

**Authors:** Charlotte Leboeuf-Yde, Lise Hestbæk

**Affiliations:** 1 Spinecenter of Southern Denmark, Hospital Lillebaelt, Østre Hougvej 55, DK-5500 Middelfart, Denmark; 2 Institut Franco-Europeen de Chiropratique, 24, Boulevard Paul Vaillant Couturier, F-94200 Ivry-sur-Seine, France; 3 Nordic Institute of Chiropractic and Clinical Biomechanics, Forskerparken 10, DK-5230 Odense M, Denmark

## Abstract

Many health science research and review articles end with the words: "More research is needed". However, when it comes to research, it is not as much a question of quantity as of quality. There are a number of important prerequisites before research should be initiated. The three pillars, relevance, quality and ethics should be respected but for a project to be meaningful, it must also be based on plausible rationale.

In evidence-based (informed) practice, one takes into account not only research-based evidence but also clinical expertise and the patients' perspectives. In this paper, we briefly discuss how this should be handled in clinical practice is briefly discussed, using the concept of "traffic lights" (red, yellow, green). We explain how the combination of evidence and plausibility can be used to reach a decision as to whether a treatment or diagnostic procedure is suitable, possible, or unsuitable.

In this thematic series of Chiropractic & Osteopathy a number of reviews are presented, in which the research status of pediatric chiropractic is scrutinized and found wanting. Two important aspects were studied in these reviews: the effect of treatment and safety issues. Two types of problems were identified: the lack of research in general and the lack of research using the appropriate study designs and methodology in particular. Therefore, we discuss the meager research noted in the areas of chiropractic care in children and the clinical consequences this should have. The prerequisites for "more research" are scrutinized and an example given of suitable research programs.

Finally, the important issue of implementation of research findings is covered, emphasizing the responsibility of all stakeholders involved at both the undergraduate and the postgraduate level, within professional associations, and on an individual level.

## Introduction

### Always more research?

Many health science research and review articles end with the words: "More research is needed". However, when is more research really needed? Obviously, research should be relevant, of good quality and ethically defensible.

## Discussion

### When is research relevant?

Whether research is relevant or not is decided by the researchers and by the funding bodies, who are willing to pay for it. Relevance is often considered on the basis of one or several of the following factors: the incidence/prevalence of a problem, its seriousness/importance and its associated costs or consequences.

When conducting health-related research on people, there must also be an anatomical/physiological/biological rationale. Sometimes there is a previous study indicating the way to go, the clinical experience points in a certain direction, or new information may challenge a previous view. If none of these criteria are present, the study subject does not appear relevant.

### When is research of good quality?

Providing that the study is considered relevant, how does one then assure sufficient quality? The most important aspect to the quality of research is to choose a *study design *that is able to bring forth an answer to the research question. For example, if you want to know if treatment A "works" or not, it is necessary to compare treatment A with treatment B or with no treatment at all and to do this in a randomized controlled clinical trial (RCT). If your question is in relation to the cause of a disease, it is necessary to compare people subjected to the suspected cause with those not subjected to it. To investigate if a clinical test is useful, it is necessary to be compare how the test behaves in both diseased and non-diseased people and, if possible, to compare its accuracy to a "gold standard" test.

Having selected the correct study design, it is thereafter important to comply with a set of rules for the *methods *used in the study. These rules differ depending on the study design. There are a number of check-lists that can be consulted and used when planning such studies [1-3]. If the researcher does not follow these rules, the data are likely to be flawed and therefore the end result cannot be trusted. For example, when studying the effect of treatment, it would be necessary to randomly allocate study subjects into different treatment groups and to investigate the outcome without having the possibility of something other than the treatment to influence the results, either on purpose or subconsciously. In the publication process, a lot of importance is placed on the quality aspect of the research and of the research report. The quality control is taken care of during the peer-review, providing that the peers who do the reviewing are sufficiently knowledgeable and thorough to assume this task.

Unfortunately, some studies of poor quality are published. Such publications can cause considerable damage, as the uninformed reader could be fooled into believing in the study results, regardless of whether they are credible or not.

### When is research ethically defensible?

Ethics covers both care and respect for study subjects. Everybody will surely agree that research should not be harmful to study subjects. It may not be quite so obvious that it is also unethical to conduct irrelevant studies or studies of poor quality. If the study turns out to be superfluous, if the study design is such t hat it cannot bring an answer to the research question, or if the method is such that the answers are not trustable, the study is unethical. In more detail, these reasons are as follows: 1) it is not fair to inconvenience study subjects with studies of no consequence, 2) poor quality studies draw limited research funds away from studies of higher quality, and, very importantly, 3) if published they can mislead clinicians who trust in research and believe in what they read.

### What can research be used for in clinical practice? The three legs of evidence-based practice

The essence of the decision making process for responsible patient care in evidence-based practice is that it relies on the integration of 1) clinical expertise,2) patient perspectives, and 3) the best evidence (i.e. research findings) [4]. However, it is important to keep a humble attitude to one's own clinical experience and not to think that it overrides the evidence obtained in a good quality RCT. Also, one or even several persons' clinical experience is not synonymous with clinical expertise.

### When are we "allowed" to treat our patients?

In chiropractic practice, if there is high quality research evidence in favor of a clinical treatment, this constitutes a *green light for *action.

If there is no research evidence, the first two legs of the evidence-based model, experience and the patient perspectives, may still justify treatment. This corresponds to the *yellow light*, where one can proceed with caution under certain circumstances. Obviously, it would not be suitable to *claim *to be able to treat untested conditions. But if approached by a patient who would like to try such a treatment, it may be acceptable to provide a trial of treatment, providing that the treatment is safe, and that the attempt is interrupted if no obvious changes occur within a brief period of time.

However, if the treatment lacks anatomical/physiological/biological plausibility, treatment is never acceptable. The same is the case, if there is evidence working against a treatment. These are to be considered obvious *red lights*.

### Which diagnostic tests can we trust?

Any treatment depends on the diagnosis. Evidence that a diagnostic procedure or system actually helps separate the sick from the healthy or that it makes a difference in relation to treatment outcome, turns the diagnostic procedure into a *green light*-procedure; one that can be used with good confidence.

Unfortunately only few diagnostic tests relevant to chiropractic practice have been tested or tested properly. If the test is biologically plausible but untested, it corresponds to a *yellow light *and should not be used as the sole rationale for treatment. However, it can be used in conjunction with other plausible information to help form an overall opinion of the case.

If the test is neither biologically implausible nor validated, it corresponds to a *red light *situation and should not be used.

### Chiropractic care and children

Over the past years there has been some controversy about whether chiropractors can treat children or not. The question appears nonsensical. Why should chiropractors not be able to treat children? Obviously the scope of complaints will differ somewhat for different age groups, and the management strategy including choice of treatment methods will have to be adapted to the individual, but this is not specific to children. This argument seems to be supported by the fact that when children are treated by chiropractors the way adults are treated in chiropractic practice (i.e. mainly for musculoskeletal disorders and for the occasional "other" condition), no such controversy appears to exist.

Rather, the source of the controversy seems to be various claims of miracle cures of "other" conditions, such asthma, enuresis, otitis media, colic, and attention deficit/hyperactivity disorder. Such information sometimes appears on chiropractors' homepages or at seminars, often masked as evidence-based fact. When such claims are ill-founded, as the reviews in this theme series of Chiropractic & Osteopathy show them to be, it is not surprising that they cause ill-feelings.

Whenever there is controversy as to whether a treatment is suitable or not, we should turn toward the research literature to find the hard evidence available. Anecdotes, case studies, ideology, feelings and subjective arguments do not form a sufficient basis on which treatment can be based.

### Present level of research evidence

In this theme series of Chiropractic & Osteopathy a number of reviews are presented, in which the research status of pediatric chiropractic is scrutinized and found wanting. Two important aspects were studied in these reviews: the effect of treatment and safety issues. The conclusions can be summarized in this one sentence. Two types of problems were identified: the lack of research in general and the lack of research using the appropriate study designs and methodology in particular.

### Positive effect of treatment

Generally, the reviews tell the same story in relation to effect of treatment of children regardless the area that was under review: absence of suitable well designed RCTs and, at best, conflicting evidence. However, absence of evidence of effect is not the same as evidence of absence of effect [5] and this absence of facts is not unique to chiropractic. Due to limited resources, both in terms of finances and manpower, most treatments of the various conditions in childhood are still scientifically untested. However, this is not generally considered a large problem, as long as the treatment is based on generally accepted basic science knowledge and logical treatment models.

### Adverse effects

Any treatment for a disorder can be expected to have a) a positive effect b) no effect, or c) an adverse effect, or a combination of these. When studying positive treatment effects, the only generally accepted study design is that of an RCT. In the absence of an untreated control group, one can only observe outcome, which may or may not be caused by the therapy provided.

Interestingly, when studying adverse effects of spinal manipulation, simple case-reports or series of case-reports are apparently considered sufficient to prove *effect *(in this case: adverse effects) of treatment. In other words, studies with an improper design appear to have been considered acceptable and the mere presence of temporality (treatment occurred before the adverse event) has been used as evidence of cause-effect.

Severe complications after spinal manipulations are rare [6,7] and therefore unsuitable for study in an RCT, because such a study would have to contain millions of patients before firm conclusions could be drawn as to whether specific complications could be the product of spinal manipulation. Other more tricky designs, such as case-control studies, preferably from large population-based registers will have to be used instead. Given the relatively small number of children treated in chiropractic practice and the difficulty in conducting such studies, it is unlikely that in the short term we will obtain such information on the pediatric population.

However, according to Humphreys [7], over the past 50 years or so, only 54 cases of adverse reactions have been recorded after chiropractic care in patients under the age of 18, and most of these were not serious in nature or not directly related to the treatment. This area, therefore, does not seem to be of highest research priority, as it does not fulfill any of the relevance criteria (common, severe, costly). Setting a large research program en route would be a bit like sailing out in an armada looking for the Loch Ness monster. This, of course, does not free the chiropractic practitioner from the duty to ensure that all treatments, also of children, are provided only on clear indications ("Is it logical?", "Has it been shown to work?"). In addition, in the case of trial treatments, it is important to terminate the treatment if no clinically detectable results are obtained. In fact, treatments without a plausible rationale should never be given, because the possibility of an unsuitable reaction is always possible.

### "More research is needed"

As our resources are limited, it is necessary to consider carefully what research it we need more of. Common, severe and/or costly conditions with a plausible scientific rationale should of course be highest on the list of priorities. Back pain seems to be an obviously relevant research area, as it is a condition that starts early in life [8], and once there, has a strong tendency to recur later in life [9]. It is costly [10] and, for some people, although not life-threatening, can have considerable consequences for some [11]. The causes of back pain or non-recovery from back pain are not well understood, and it is a condition commonly treated by chiropractors [12,13]. RCTs have shown that spinal manipulation in patients with back pain in some cases will have an effect [14]. There are several plausible proposed explanations for why this could happen [15,16]. In sum, back pain is a good example of a suitable topic for a research program in children. Such a program is described in Appendix 1.

From the large array of research topics proposed in Appendix 1, it becomes clear that data should be collected in many different ways, such as through epidemiologic studies, various registers, in everyday practice and through laboratory experiments. Data can also be collected by asking questions, observing, and using advanced technology. Importantly, the research design should match the research question and the conclusion should not exceed the result.

In order to set up a research program on chiropractic treatment of "other" conditions in children, similar criteria for relevance may be more difficult to fulfill, particularly that of plausibility. Also, it would be necessary to consider why this condition should be studied only in childhood, as it is unclear whether there is some age-limit beyond which chiropractic care of these conditions no longer has an effect. For example, some plausible reason why ADHD can be treated in children but not in adults should be stated.

### Fewer case-reports are needed

When calling for more research, it must be understood that this excludes case-reports. Case-reports are usually easy to read, bring mainly clinical elements into the picture, tell an unusual story, are often accompanied by photographs, and seem to be very attractive to clinicians. However, they are rarely representative of what typically happens in clinical practice.

Further, when they describe a positive outcome, this cannot be interpreted as proof of treatment effect for, at least, the following reasons: First, improvement after treatment may not be causally linked to the treatment; it could have happened anyway. Second, one treated case may well have improved with treatment but, because none of the other similar patients who did not recover were described, the relative importance of this phenomenon cannot be estimated. Third, because case-reports are often retrospective, data collection was not geared towards accurate recording. For the same reason, there would have been no quality control of the examination, treatment, or write-up. Fourth, the outcome would have been reported directly to the clinician, and both patient and clinician would have been willing to see a positive effect, thus the outcome measurement would be subjective. In fact, case-reports have about the same draw-backs as the experience clinicians have in their own clinic.

Therefore, the research community, health care planners and policy makers are not impressed when case-reports are used as "evidence" of effect of treatment because this type of "evidence" cannot result in any general conclusions or recommendations. And, for the same reason, clinicians should be weary of their own observations.

### Implementation of research findings

More research is always needed. However, research on its own cannot transform a clinical profession. In the case of pediatrics, we can see at least three barriers to implementation.

First, there is a lot of respect for research. It is therefore not surprising that when chiropractors consult research articles they will often believe what they read, even if the research lacks credibility. Presently, most chiropractic pediatric research literature lacks credibility. When editors of second-rate scientific publications and some lecturers at professional seminars pretend to provide an evidence-based treatment model, clinicians may not realize that the new treatment method is, in fact, largely imagination-based.

Second, most clinicians, including chiropractors, have little time to read research papers, and if they do, they are often uncertain of the quality of the research. Therefore, they probably often rely on other external sources such as hearsay, information obtained at seminars, and simple professional texts such as case-reports. If this information is grossly incorrect, they will practice imagination-based chiropractic but in good faith.

Third, clinicians, who practice in a lucid mainstream manner, may change approach in relation to children, because sick children are vulnerable, parents demand treatment and expect cure, and it is difficult to ignore a suffering child. They may therefore seek to help at all costs, perhaps forgetting that most of these "other" conditions are self-limited or transitory. However, it is not in good taste to play on the vulnerability of others who are easy victims of bogus claims. This is true, whether the victims are clinicians attending a seminar or patients seeking care.

With more relevant and high quality research, with better and more user-friendly information to clinicians, and with more vigilance from the lecturers in undergraduate programs, presenters at professional seminars, and professional associations, it should be possible to provide patients of all ages with relevant and effective treatment also when they attend a chiropractor's office. Nevertheless, the final responsibility is still in hands of the individual practitioner.

## Conclusion

We believe that the answer to the question proposed in the title "Is more research enough?" is "No". We must not be satisfied with quantity but strive towards high standards of quality of the published research and work harder at all levels on implementing research into practice.

## Authors' contributions

CL conceived of the study and made the first draft. Both authors contributed to the manuscript and approved the final manuscript.

## Appendix 1. Example of three research programs on back pain in children

1. The course and character of back pain:

• Onset/continuation/development, symptoms of spinal pain from the early years.

• Development over age for various spinal disorders

• The association between early spinal degeneration and spinal pain

• Normal and abnormal pain patterns of symptoms

2. Causes of back pain

• The respective roles of genetic and extrinsic factors in both onset and continuation of back pain

• The anatomical and neurophysiological mechanisms for the maintenance of and the recovery from spinal symptoms

• The role of intrinsic and extrinsic biomechanical factors in both onset, continuation and termination of back pain

3. Treatment of back pain

• Treatment response of different treatment methods

• Treatment response at different ages
